# Prontosil in Obstetrics

**Published:** 1937

**Authors:** H. J. Drew-Smythe

**Affiliations:** Obstetrician and Gynæcologist to the Bristol General Hospital; Professor of Obstetrics, University of Bristol


### PRONTOSIL IN OBSTETRICS.

BY

H. J. Drew-Smythe, M.S., F.R.C.S., F.C.O.G.,

*Obstetrician and Gynæcologist to the Bristol General Hospital; Professor of Obstetrics, University of Bristol.*

---

My experience of Prontosil in obstetrics dates from the spring of 1936, when we first used it at Southmead Hospital for cases of puerperal fever. The results were encouraging, especially in cutting short the pyrexial period. I later used it for the same purpose at Ham Green Hospital, and afterwards at the General Hospital. The improvement in the mortality rate was not obvious, as the number of cases treated was too small for comparison with those treated before the introduction of Prontosil. The most marked improvement was in the localization of the septic process and the rapidity of the cure. These initial trials convinced me of the value of this new drug, and since then I have used it in every case of puerperal fever, and for prophylaxis in cases in which septic infection is present, or intra-uterine manipulation has been carried out. The dosage varies according to the virulence of the infection. In septicæmia 15 c.cm. of Prontosil soluble is given daily for seven days, and two tablets of Prontosil album three times a day.

The amount of Prontosil album is gradually decreased as improvement takes place. In mild cases and for prophylaxis Prontosil album alone is given. The drug is discontinued when the temperature has remained normal for forty-eight hours, but repeated if there is any recurrent rise. This recrudescence of pyrexia often occurs in these cases, but is rarely severe and soon responds to treatment.

The only ill-effects of the drug which I have encountered have been the onset of sulphæmoglobinæmia and slight mental confusion with slowing of the mental processes. After administration of prontosil over a long period methæmoglobin is formed which gives a cyanosed appearance to the whole body, and is easily seen on examination of the finger nails. This is a combination easily broken down and disappears in a few days on cessation of the drug. If, however, sulphates of any kind are administered at the same time a very stable combination, sulphæmoglobin, is formed, which shows itself in the same manner but may take weeks to clear up.

Some authorities push the drug even to the production of cyanosis, and maintain this, stating that no harm results.

The majority of the patients treated showed the presence of streptococcus hæmolyticus, either on examination of the lochia or on blood culture.

In these streptococcal infections the best results were obtained. Prontosil is not specific in its action against the streptococcus. Good results have been reported in cases of *bacillus coli* pyelitis, and also in cases of staphylococcal breast infection. Its action is obviously not that of a bactericide, but probably renders the infecting organisms more vulnerable to attack by the natural defence forces.

As an example of the result and possible ill-effects of treatment with Prontosil I quote the following case :—

Mrs. P., primipara, aged 39, was admitted to the Bristol General Hospital with obstructed labour and failed forceps delivery. On examination the presentation was found to be a face with posterior rotation of the chin. Delivery by rotation and forceps was difficult, but eventually accomplished with the birth of a still-born child. Labour was further complicated by an adherent placenta, but this was expressed without having to resort to manual removal. On the fourth day of the puerperium she developed a septicæmia accompanied by rigors and rapid pulse. Prontosil album three tablets (gr. v) t.d.s. had been given from the first day of the puerperium and continued in this dose throughout the course of the illness. After fourteen days the temperature abated, but the patient was showing considerable cyanosis, rapid pulse and respiration, and mental dullness. The administration of Prontosil was stopped immediately. The blood showed definitely the presence of sulphæmoglobin. The cyanosis very slowly improved, but her mental condition became slightly worse, and nineteen days later she was transferred to a mental hospital with a diagnosis of toxic psychosis, from which she eventually made a complete recovery.

I do not think that one can definitely attribute the mental condition to the effects of the administration of Prontosil, except that the patient did not manifest the ordinary symptoms of puerperal mania; and I have seen mild degrees of psychosis in other cases where Prontosil has been administered. In spite of these possible complications I am firmly convinced that in Prontosil and its derivatives we have a most potent drug at our disposal in preventing the morbidity and mortality associated with puerperal infection.

